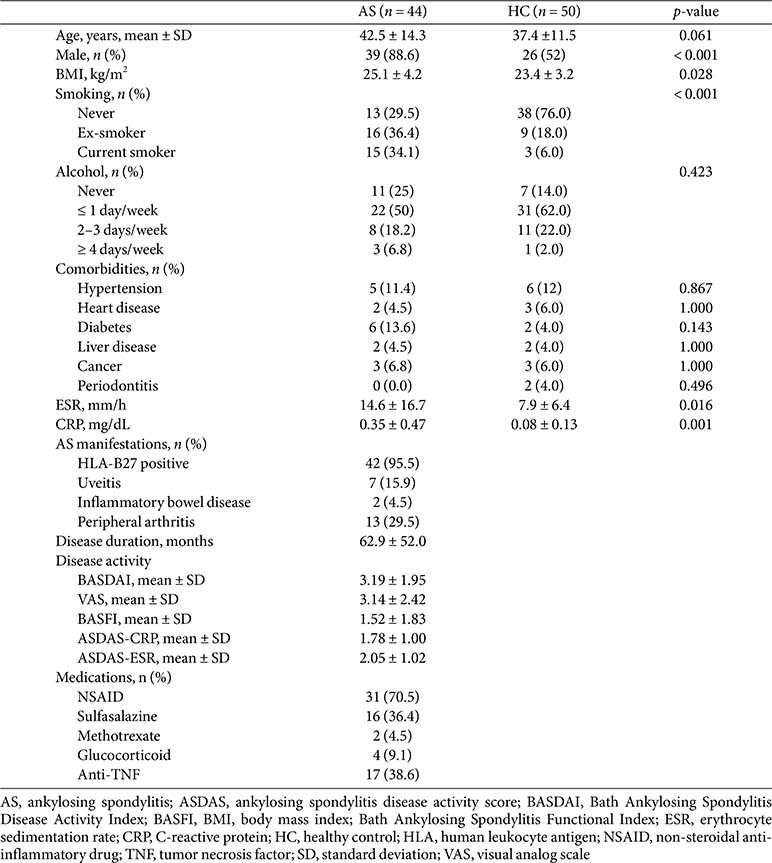# Alterations in the Gut Microbiome in Ankylosing Spondylitis and Their Correlation with Disease Activity

**DOI:** 10.4014/jmb.2508.08043

**Published:** 2025-12-18

**Authors:** Hyemin Jeong, Hoonhee Seo, Sukyung Kim, Md Abdur Rahim, Indrajeet Barman, Md Sarower Hossen Shuvo, Sujin Jo, Mohammed Solayman Hossain, Jeong-Ju Yoo, Young Ho Kim, Sung-Soo Jung, Ho-Yeon Song, Chan Hong Jeon

**Affiliations:** 1 Department of Internal Medicine, Soonchunhyang University Bucheon Hospital, Bucheon 14584, Republic of Korea; 2 Department of Microbiology and Immunology, School of Medicine, Soonchunhyang University, Cheonan-si 31151, Republic of Korea; 3 Human Microbiome Medical Research Center (HM·MRC), School of Medicine, Soonchunhyang University, Asan-si 31538, Republic of Korea; 4 Department of Urology, Soonchunhyang University Bucheon Hospital, Bucheon 14584, Republic of Korea

**Keywords:** Ankylosing spondylitis, gut microbiota, dysbiosis, biomarker, *Coprobacter*

## Abstract

The microbiome significantly influences immune dysfunction and gut dysbiosis in patients with ankylosing spondylitis (AS). This study focuses on defining the distinct microbial characteristics within AS and biomarkers associated with disease activity. 44 patients with AS and 50 healthy controls (HC) were recruited. 16S rRNA sequencing was conducted to assess the microbiome of stool samples. The Ankylosing Spondylitis Disease Activity Score with C-reactive protein (ASDAS-CRP) was calculated for all AS patients; scores ≥ 2.1 indicated high disease activity, while < 2.1 indicated low disease activity. Similar alpha diversity profiles were maintained in both AS and HC cohorts, whereas significant differences were identified in beta diversity. The compositional prevalence of Proteobacteria, particularly Gammaproteobacteria, and Enterobacterales, including *Escherichia* spp., in the AS group was significantly increased. On the other hand, beneficial taxa, including Firmicutes, Clostridia, Clostridiales, Lachnospiraceae, Ruminococcaceae, and *Faecalibacterium*, were highly abundant in the HC group. Among patients with AS, alpha diversity decreased in the high disease activity group compared to the low disease activity group, while beta diversity did not differ significantly. Moreover, *Coprobacter* spp. abundance positively correlated with the Bath Ankylosing Spondylitis Disease Activity Index (*p* = 0.032) and the ASDAS-CRP (*p* = 0.023). Patients with AS exhibit distinct gut microbiota profiles, with increased Proteobacteria and decreased beneficial taxa such as Firmicutes. Greater disease activity is accompanied by reduced alpha diversity, while *Coprobacter* spp. abundance correlates with disease activity, suggesting its potential as a biomarker.

## Introduction

Spondyloarthritis (SpA) is a chronic inflammatory disease primarily affecting the axial skeleton, peripheral joints, and entheses. Additionally, it can be accompanied by extra-articular symptoms, for example, uveitis, psoriasis, as well as inflammatory bowel diseases (IBD), namely Crohn's disease and ulcerative colitis. Ankylosing spondylitis is the most common form of SpA and is characterized by radiographic changes in spine and sacroiliac joints. Approximately 5–7% of AS patients have IBD [1], while 50% have microscopic gut inflammation [2]. Moreover, heightened gut inflammation corresponded with a greater extent of bone marrow edema in the sacroiliac joints [3] and with progression from non-radiographic axial spondylitis to AS [4]. Hence, AS pathogenesis is closely linked to the gut.

Genetic factors are closely linked to AS development, with more than 90% of patients carrying the human leukocyte antigen (HLA)-B27 gene [5]. Misfolding of HLA-B27 induces endoplasmic reticulum stress and activates the unfolded protein response, enhancing interleukin (IL)-23 production and contributing to bowel inflammation [6]. IL-23 drives the progression of colitis by upregulating downstream proinflammatory effectors, such as IL-17, IL-6, and TNF-α [7]. Moreover, IL-23 promotes the development of spondyloarthropathy by stimulating ROR-γt^+^CD3^+^CD4^−^CD8^−^ enthesial resident T cells [8].

Gut dysbiosis is also common in patients with AS, suggesting that the microbiome engages in a complex interplay with genetic and immune-mediated dysfunction. HLA-B27 contributes to alterations in the gut microbiome [9], as evidenced by arthritis and colitis development in over 80% of HLA-B27 transgenic rats with commensal microbiota [10]. Even healthy HLA-B27-positive individuals without disease exhibit intestinal microbiota alterations [11]. The inflamed gut, influenced by microbiota, is a key site of IL-23 production [12] and the activation and expansion of innate lymphoid cells (ILCs), which accumulate in AS-inflamed areas [13, 14]. Indeed, differences in the intestinal microbiota of AS patients compared with healthy controls have been reported; however, the results have been inconsistent [15-18]. Nevertheless, dysbiosis has been linked to AS activity, with *Dialister* relative abundance in colonic biopsies correlating positively with the Ankylosing Spondylitis Disease Activity Score (ASDAS) [19]. Similarly, *Ruminococcus gnavus* in fecal samples is associated with the Bath Ankylosing Spondylitis Disease Activity Index (BASDAI) in patients with SpA [20]. However, research on taxonomic biomarkers for AS activity is limited.

The research objectives were to identify distinct microbial characteristics and biomarkers correlated with disease progression in AS patients.

## Methods

### Study Population

Patients with AS were recruited from the Rheumatology Outpatient Clinic at Soonchunhyang University Hospital, Bucheon, South Korea, between December 2022 and December 2023. Inclusion criteria included age ≥ 19 years, fulfillment of updated New York criteria of AS [21], as well as agreement to participate in the study. A total of 44 patients were enrolled in the study. The healthy control group comprised 50 randomly recruited individuals through in-hospital advertisements during the same period. All participants were recruited from a single center in Korea and were of Asian (Korean) ethnicity. The eligibility criteria for the HC group included age ≥ 19 years and absence of autoimmune diseases, such as rheumatic diseases or IBD.

### Data Collection

Demographic information, including age, sex, alcohol consumption, smoking status, and comorbidities, was collected using a self-assessment questionnaire. Medication history was extracted from patients’ medical records, including key clinical features of AS, such as uveitis, IBD, and peripheral arthritis. HLA-B27 results obtained as part of routine diagnostic testing during visits to the rheumatology clinic were also recorded. Disease activity was evaluated using the BASDAI [22], Bath Ankylosing Spondylitis Functional Index [23], and Visual Analog Scale (VAS) for overall patient condition, with scores from 0 to 10. Additionally, erythrocyte sedimentation rate (ESR) and C-reactive protein (CRP) levels were determined. The ASDAS was calculated based on these results. An ASDAS-CRP score ≥ 2.1 constituted AS with high disease activity (ASH), while a score < 2.1 indicated AS with low disease activity (ASL).

### Genomic DNA Extraction from Stool Samples

Fecal samples were collected from all participants, frozen immediately, followed by storing at -80°C. The QIAamp DNA Fast Stool Mini Kit (Qiagen, Germany) was utilized for stool DNA isolation, implementing the recommended procedures. The quantification of DNA was done with a Fluorometer (Qubit4, Thermo Fisher Scientific, USA), and integrity of DNA was evaluated by gel electrophoresis (agarose 0.8%) and subsequently preserved at −20°C till subsequent analyses. Through all procedural iterations, DNA samples were measured for concentration, standardized, and assessed for quality, including DNA extraction, metagenomic library creation, and sequencing. Sterile conditions were maintained for all processes, and template-free controls were used.

### 16S rRNA Gene Amplification and Metagenomics Library Preparation

Bacterial DNA extracted from the fecal samples was subjected to PCR amplification of the V4 hypervariable region of the 16S rRNA gene. PCR amplification was conducted using a Veriti 96-well Thermal Cycler (Applied Biosystems, Thermo Fisher Scientific) under Illumina’s recommended amplicon PCR conditions along with negative control. Negative controls (blank extractions and PCR blanks) were included to check for contamination. Each reaction mixture (25 μl) contained 5 μM of reverse and forward primers, template DNA of 10 ng, DNase-free water and KAPA ReadyMix (Kapa Biosystems, USA) [24]. The primers used for 16S rRNA gene amplification were previously reported [25]. After PCR, the amplicons were purified using AMPure XP beads (Beckman Coulter, UK) to remove unwanted fragments and contaminants. The Nextera XT DNA Library Prep Kit (Illumina, USA) was used for index PCR to incorporate unique barcodes. Indexed libraries were further purified, diluted to a final concentration of 1 nM within 10 mM Tris buffer, along with pooled by combining 5 μl from every individual preparation. To ensure sequence diversity, the 50 pmol pooled libraries have been spiked along 30% phiX (Illumina) before sequencing. The final library was sequenced on the iSeq100 platform (Illumina) to generate high-quality reads for downstream analysis.

### Bioinformatics Analysis Based on 16S rRNA Gene Amplicons

The iSeq100 Illumina FASTQ reads were examined using QIIME 2 2021.11 [26]. Sequences were identified, corrected, and filtered according to their quality using the q2-demux plugin. The data were denoised using DADA2 [27]. The QIIME 2 command line eliminated characteristics that appeared fewer than five times. A phylogenetic tree based on q2 phylogenies was constructed using fasttree2, and distinct amplicon sequence variations (ASVs) were aligned using Mafft [28, 29]. Comprehensive sequencing metrics were performed, including read counts, quality filtering findings, and ASV data following our previous publication [30]. Sequences were assigned to operational taxonomic units (OTUs) with a 97% similarity. Individual OTUs were identified by their corresponding exemplary sequences, to which the RDP classifier assigned taxonomic information. In accordance with the Human Microbiome Database, the representative sequences were categorized into different taxonomic levels, *i.e.*, phylum to species. Using a 97% cut-off parameter, a Bayesian approach was used. In order to understand the bacterial heterogeneity, sampling-based OTU analysis was conducted; this was reported as a rarefaction curve. For alpha diversity, species richness was estimated using the ACE, Chao, and Jackknife indices and operational taxonomic unit (OTU) counts [31-33], whereas the NPShannon, phylogenetic diversity, Shannon, and Simpson indices were used to assess species diversity [34-36]. Beta diversity has been computed using Bray–Curtis, Jensen–Shannon, UniFrac and Generalized UniFrac distances, besides statistical significance was calculated through PERMANOVA [37-40]. ASVs were classified by alignment with the Greengenes reference database (version 13-8) at various taxonomic levels [41]. For some taxonomic classifications, particularly at the genus level, the Greengenes reference database uses suffixes like _g23 to distinguish between different clusters of the same genus. The METAGENAssist web service was used to examine the taxonomic composition [41]. To identify taxonomic biomarkers, statistical comparisons were conducted using the Kruskal–Wallis H tests and linear discriminant analysis effect size (LEfSe) (α = 0.05, LDA cutoff = 2.0) [42, 43].

### Statistical Analysis

All statistical analyses were performed with a significance threshold of *p* < 0.05. Alpha diversity was evaluated using richness (ACE, Chao, Jackknife, OTU counts) and diversity indices (NPShannon, Phylogenetic diversity, Shannon, Simpson), while beta diversity was assessed by PERMANOVA based on Bray–Curtis, Jensen–Shannon, UniFrac, and Generalized UniFrac distances [44]. Taxonomic differences at multiple levels (phylum to genus) between HC and AS groups, as well as ASL and ASH disease activity groups, were tested using the Wilcoxon rank-sum test, with *p* < 0.05 and *p* < 0.01 denoted as * and **, respectively. Microbial biomarkers were identified using the Kruskal–Wallis H test followed by LEfSe, which integrates non-parametric testing with effect size estimation to control for multiple comparisons. Finally, correlations between bacterial genera and clinical indices (CRP, BASDAI, ASDAS-CRP) were analyzed using Pearson’s correlation coefficient.

## Results

### Characteristics of the Study Population

There was no significant difference in age between AS and HC (Table 1). The proportion of male patients in the AS group was significantly higher than in the HC group. The proportion of current smokers was significantly higher in the AS group than in the HC group. There were no significant differences in comorbidities between the groups. Among the AS patients, seven (15.9%) had uveitis and two (4.5%) had inflammatory bowel disease. Additionally, seventeen (38.6%) AS patients were treated with anti-TNF agents; no participant used anti-IL-17 antibodies.

### Comparative Analysis of the Gut Microbiome between the HC and AS Groups

**Fig. 1:**
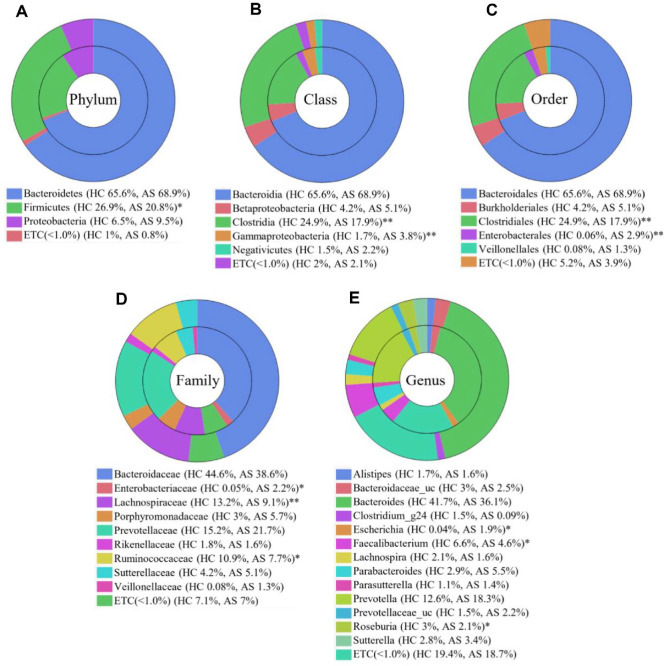
Average taxonomical composition for AS disease between the HC and AS groups. Taxonomic relative abundance at the (**A**) phylum, (**B**) class, (**C**) order, (**D**) family, and (**E**) genus levels; relative abundances < 1% are expressed as ETC. Wilcoxon rank-sum test was used to analyze the significance between the two groups. *p < 0.05; **p < 0.01; outer circle: HC group, inner circle: AS group; AS: ankylosing spondylitis, HC: healthy control.

The average composition of taxa in gut microbiota in the HC and AS groups was examined at the phylum, class, order, family, and genus levels. Only taxa with relative abundance > 1% and significant differences were summarized (Fig. 1). At the phylum level, Proteobacteria (HC = 6.5%, AS = 9.5%) and Bacteroidetes (HC = 65.6%, AS = 68.9%) showed higher abundance in the AS group compared to the HC, whereas they showed no significance (Fig. 1A). Meanwhile, Firmicutes, comprising a predominant share of the microbiome in HC group (HC = 26.9%, AS = 20.8%), while in AS was significantly reduced (*p* = 0.017). Gammaproteobacteria, a subgroup of Proteobacteria, was significantly higher in the AS group at the class level (HC = 1.7%, AS = 3.8%, *p* = 0.008; Fig. 1B). Meanwhile, Clostridia, a major class within Firmicutes, was significantly reduced in the AS group (HC = 24.9%, AS = 17.9%, *p* = 0.005). At the order level, Enterobacterales abundance significantly increased in the AS group (HC = 0.0%, AS = 2.9%, *p* = 0.006; Fig. 1C). Clostridiales, similar to its parent class Clostridia, was significantly reduced in the AS group (HC = 24.9%, AS = 17.9%, *p* = 0.005). At the family level, Lachnospiraceae and Ruminococcaceae were also significantly decreased in the AS group (HC = 13.2%, AS = 9.1%, *p* = 0.001, and HC = 10.9%, AS = 7.7%, *p* = 0.031, respectively; Fig. 1D); Enterobacteriaceae, which includes potential opportunistic pathogens, exhibited a significant elevation in AS group (HC = 0.0%, AS = 2.2%, *p* = 0.010). At the genus level (Fig. 1E), *Escherichia*, a member of Enterobacteriaceae, was significantly higher in the AS group (HC = 0.0%, AS = 1.9%, *p* = 0.034), while *Roseburia* was significantly decreased (HC = 3.0%, AS = 2.1%, *p* = 0.019). Likewise, *Faecalibacterium* was noticeably lower in AS group (HC = 6.6%, AS = 4.6%; *p* = 0.013). Overall, the taxonomic composition suggests that the gut microbiota of the AS group exhibits a decrease in bacterial groups such as Lachnospiraceae, Ruminococcaceae, and *Faecalibacterium* and an increase in taxa such as Enterobacteriaceae and *Escherichia*.

### Microbiome Diversity and Cluster Assessment in the HC and AS Groups

**Fig. 2:**
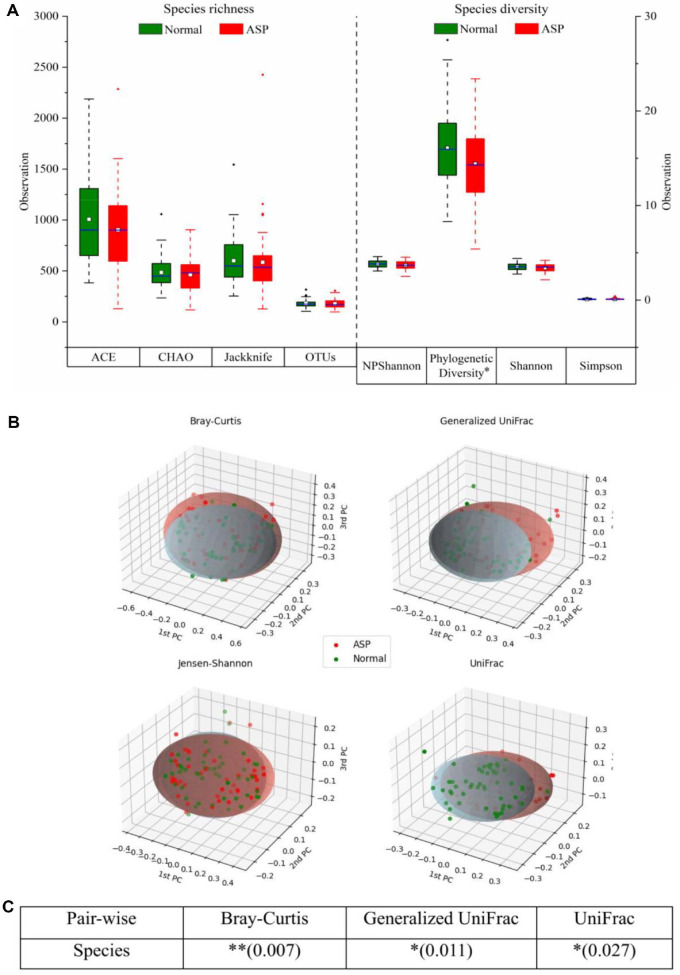
Alpha diversity and beta diversity indices for the HC and AS groups. (**A**) Species richness assessed based on Ace, Chao, and Jackknife indices and operational taxonomic units (OTUs); species diversity assessed based on NPShannon, Shannon, and Simpson indices and Phylogenetic diversity (*p < 0.05); boxplot edges denote the first and third quartiles; thick horizontal blue band: median values. (**B**) Principal coordinate analysis (PCoA) of the distance between communities. (**C**) Beta set significance was assessed using a permutational multivariate analysis of variance (PERMANOVA). AS: ankylosing spondylitis, HC: healthy control.

To evaluate microbial richness and diversity, alpha diversity was examined between the HC and AS groups (Fig. 2A). ACE, Jackknife, Chao and OTUs were used to evaluate species richness which estimated total number of OTUs while accounting for rare species. Preliminary analyses, particularly the ACE and Jackknife indices, indicated that the HC group exhibited higher species richness values than the AS group. In contrast, the Chao and OTU counts yielded similar values across both groups, suggesting comparable richness in certain aspects. The lower ACE and Jackknife values in the AS group may reflect disruptions in community stability and colonization patterns. Microbial diversity was assessed using the NPShannon, Phylogenetic Diversity (PD), Shannon, and Simpson indices. Among these metrics, the phylogenetic diversity was lower in the AS group than in the HC group. Nevertheless, no notable differences were observed among the two groups in the NP Shannon, Shannon, and Simpson indices. Despite the observed differences in richness and phylogenetic diversity, across any alpha diversity metrics between the two groups there was no statistical significance. For global diversity level, the gut microbiota of AS group did not exhibit drastic alterations compared to that of the HC group.

The assessment of beta diversity was done to evaluate differences in microbial community prevalence across groups based on the generalized UniFrac, Bray-Curtis, Jensen-Shannon and UniFrac distance metrics. Principal coordinate analysis (PCoA) was performed using these metrics to explore beta diversity patterns (Fig. 2B). The results demonstrated that the microbial community distribution varied significantly between groups. In the Bray-Curtis, generalized UniFrac, and UniFrac analyses, the AS group exhibited greater dispersion in PCoA coordinates than the HC group. In contrast, Jensen–Shannon analysis revealed a more uniform clustering pattern. Following normalization, the microbial diversity patterns appeared more comparable across groups, as reflected by the clustering distribution in the PCoA plots. The significance of between-group differences was determined using PERMANOVA (Fig. 2C). Pairwise comparisons revealed remarkable differences in microbial composition at the species level, as indicated by Bray-Curtis (*p* = 0.007), Generalized UniFrac (*p* = 0.011), and UniFrac (*p* = 0.027) analyses. These findings suggest that the structure of the microbial community varied significantly among the groups.

Furthermore, the AS and HC groups were classified into distinct groups using hierarchical clustering assessment on the basis of UPGMA (Fig. S1). This clustering approach was applied across multiple distance metrics, including Jensen-Shannon divergence (Fig. S1A), Bray-Curtis dissimilarity (Fig. S1B), Generalized UniFrac (Fig. S1C), and UniFrac (Fig. S1D). The results revealed no clear separation between patients with AS and HC, suggesting that microbial community structures did not exhibit distinct clustering patterns between the two groups.

### Taxonomic Biomarker Discovery (HC vs AS)

**Fig. 3:**
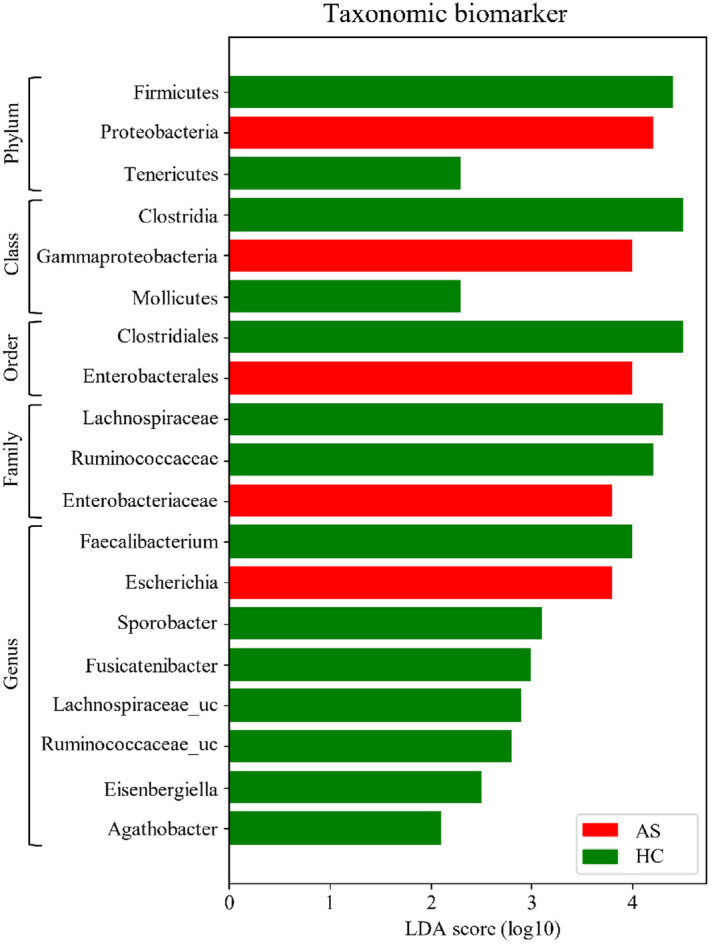
Taxonomic biomarkers for the HC and AS groups using linear discriminant analysis effect size (LEfSe). Microbiota patterns between HC and AS groups are analyzed using a linear discriminant analysis coupled with LEfSe (α = 0.05, LDA cutoff = 2.0). Taxa labels such as *Eubacterium_g23* follow the Greengenes clustering convention. Results present the most differentially abundant microbiota taxa; red: AS group, green: HC group, AS: ankylosing spondylitis, HC: healthy control.

The microbial compositional difference between the HC and AS groups have been further analyzed using LEfSe with linear discriminant analysis (LDA). The resulting forest plot highlights the most differentially abundant taxa (Fig. 3). Firmicutes was noticeably increased in the HC group at the phylum level (LDA score ≥ 4.4), whereas Proteobacteria was substantially elevated in the AS group (LDA score ≥ 4.2). At the class level, Clostridia was more abundant in the HC group (LDA score ≥ 4.5), while Gammaproteobacteria was significantly enriched in the AS group (LDA score ≥ 4.0). At the order level, Clostridiales was significantly enriched in the HC group (LDA score ≥ 4.5), whereas Enterobacterales showed higher abundance in the AS group (LDA score ≥ 4.0). At the family level, Lachnospiraceae (LDA score ≥ 4.3) and Ruminococcaceae (LDA score ≥ 4.2) have been significantly enriched in HC group, while Enterobacteriaceae has been more abundant in AS group (LDA score ≥ 3.8). At the genus level, *Faecalibacterium* (LDA score ≥ 4.0), *Sporobacter* (LDA score ≥ 3.1), and *Fusicatenibacter* (LDA score ≥ 3.0) have been significantly abundant in the HC group, whereas *Escherichia* (LDA score ≥ 3.8) was more abundant in the AS group. Additionally, *Lachnospiraceae_uc*, *Ruminococcaceae_uc*, and *Eisenbergiella* were significantly enriched in the HC group.

Clostridiales and Clostridia had the largest LDA effect sizes and were strongly associated with the HC group. Notably, *Faecalibacterium* and *Escherichia* were among the most differentially abundant genera, highlighting their potential roles as key taxonomic markers for distinguishing the two groups.

### Comparative Analysis of Gut Microbiomes between the ASH and ASL Groups

Subgroup analysis was conducted for the AS group based on disease activity. Table S1 provides an overview of the clinical features of patients with ASH and ASL. The mean body mass index of ASH was markedly higher than ASL (27.4 ± 4.2 vs. 23.9 ± 3.8, *p* = 0.006). Use of therapeutic agents, including anti-TNF drugs, did not differ significantly between the groups.

**Fig. 4:**
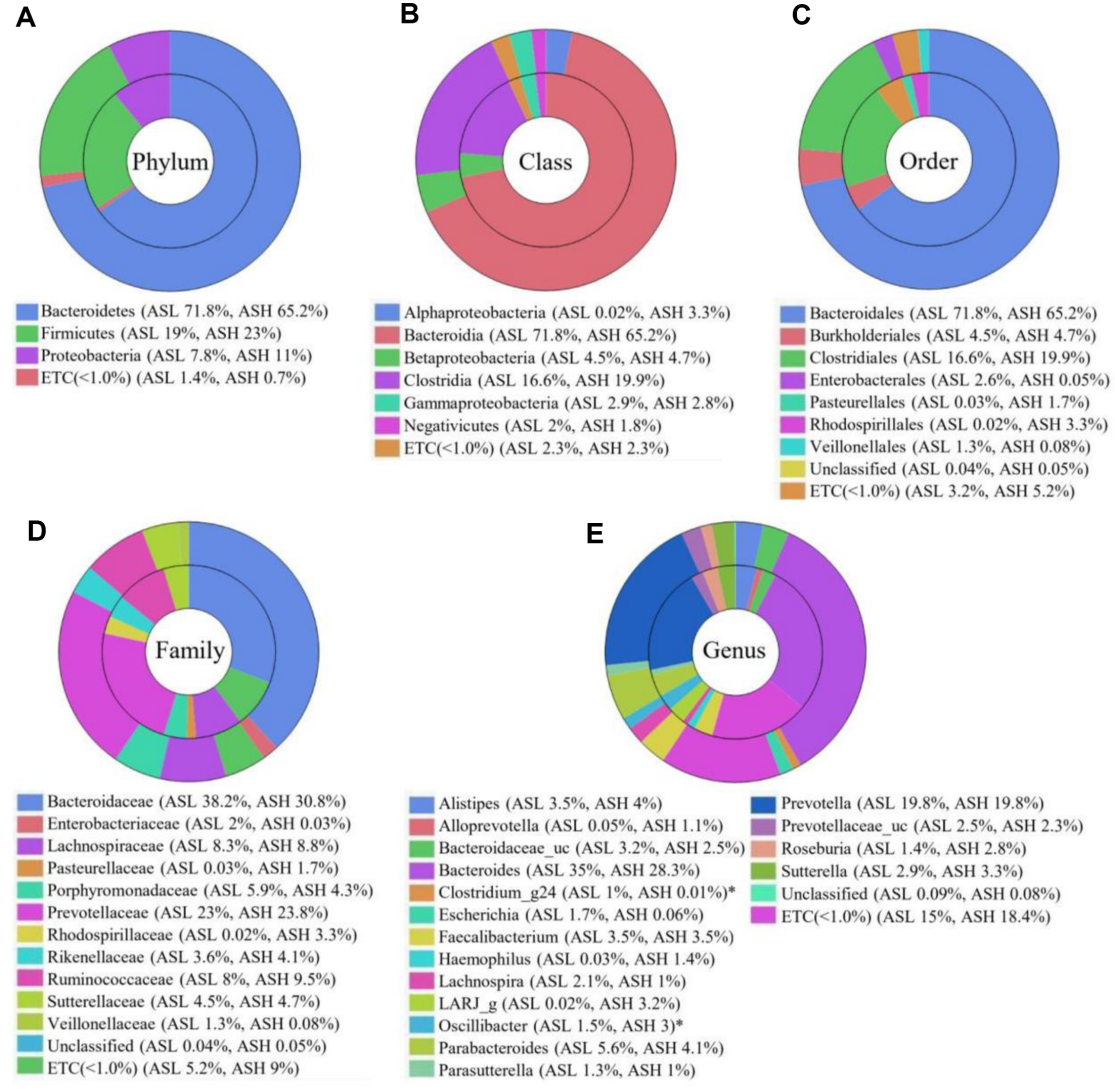
Average taxonomical composition for the ASL and ASH groups. Taxonomic relative abundance at the (**A**) phylum, (**B**) class, (**C**) order, (**D**) family, and (**E**) genus levels; relative abundance < 1% expressed as ETC. Significance between two groups assessed using Wilcoxon rank-sum test (*p < 0.05); outer circle: ASL, inner circle: ASH group. ASH: Ankylosing spondylitis with high disease activity, defined as ASDAS-CRP ≥ 2.1; ASL: Ankylosing spondylitis with low disease activity, defined as ASDAS-CRP < 2.1.

The gut microbiome compositions of the ASL and ASH groups were analyzed across multiple taxonomic levels (Fig. 4). At the phylum level, Proteobacteria was relatively more abundant in the ASH group, while the relative abundance of Bacteroidetes was lower than in the ASL group (Fig. 4A). Although Firmicutes was also slightly more abundant in ASH, the differences were notable from a statistical perspective (*p* < 0.05). At the class and order levels, the abundances of Clostridia as well as Clostridiales have been very high in the ASH group, while those for Bacteroidia and Bacteroidales were lower. Additionally, Alphaproteobacteria, Rhodospirillales, and Pasteurellales were unique to the ASH group (Fig. 4B and 4C). Bacteroidaceae and Enterobacteriaceae have been almost absent in patients with ASH, whereas Pasteurellaceae and Rhodospirillaceae were exclusively detected in these patients at family level. Whereas the comparative levels of Prevotellaceae remained stable (Fig. 4D). At genus level, *Oscillibacter* was significantly more abundant in the ASH group (ASL = 1.5%, ASH = 3%, *p* = 0.018), whereas *Clostridium_g24* was absent (ASL = 1%, ASH = 0%, *p* = 0.025). Fig. 4E). Other genera, such as *Roseburia*, *Sutterella*, and *Alistipes*, were slightly more abundant in the ASH group, while *Haemophilus* and *Alloprevotella* were unique to this group.

### Microbiome Diversity and Cluster Assessment in the ASL and ASH Groups

**Fig. 5:**
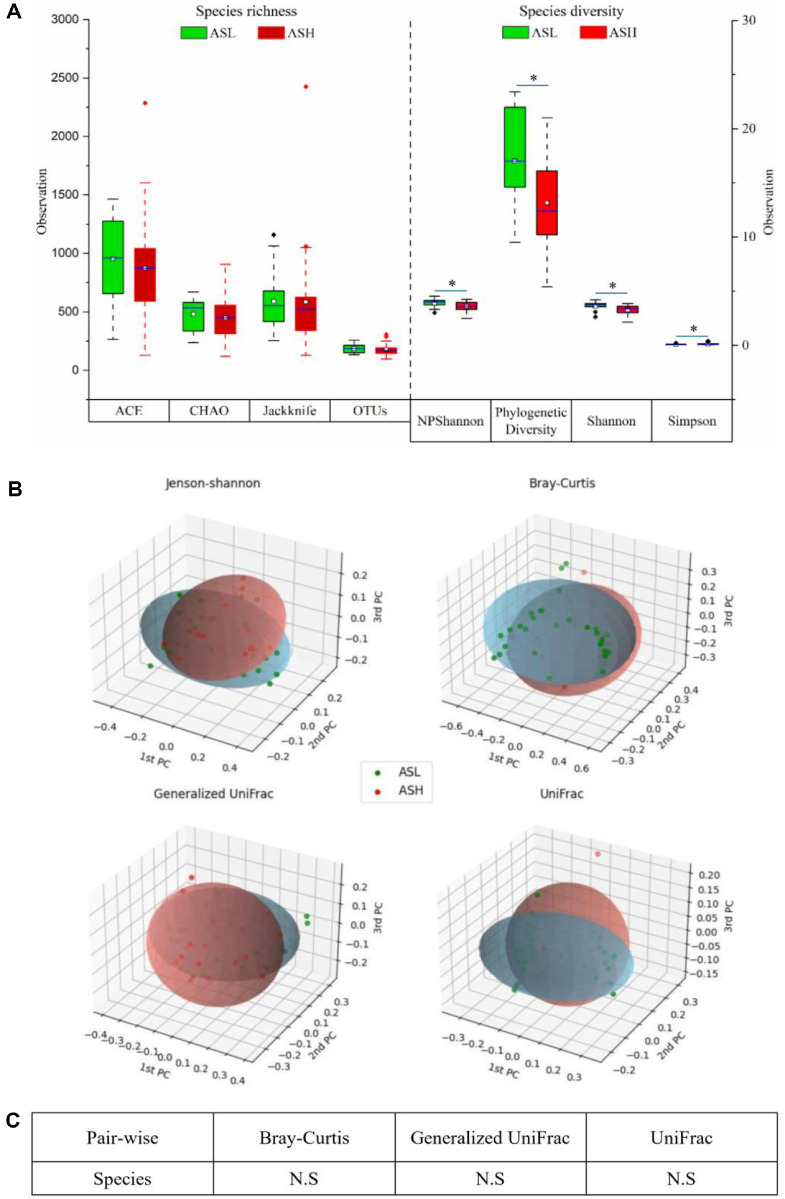
Alpha diversity and beta diversity indices for ASL and ASH groups. (**A**) Species richness assessed based on Ace, Chao, and Jackknife indices and operational taxonomic units (OTUs); species diversity assessed based on NPShannon, Shannon, and Simpson indices and Phylogenetic diversity; boxplot edges denote the first and third quartiles; thick horizontal blue band: median values. (**B**) Principal coordinate analysis (PCoA) of the distance between communities. (**C**) Beta set significance was assessed using a permutational multivariate analysis of variance (PERMANOVA). ASH: Ankylosing spondylitis with high disease activity, defined as ASDAS-CRP ≥ 2.1; ASL: Ankylosing spondylitis with low disease activity, defined as ASDAS-CRP < 2.1.

Alpha diversity was assessed to compare microbial richness and diversity between the ASL and ASH groups (Fig. 5A). The ACE, Chao, and Jackknife indices showed relatively higher species richness in ASL group compared to ASH. On the other hand, OTU counts revealed comparable richness across both groups. The differences in richness indices between the groups suggest disruptions in the microbial colonization patterns.

Microbial diversity was further evaluated using the NPShannon, PD, Shannon, and Simpson indices. The Phylogenetic Diversity index indicated a significantly higher diversity (*p* < 0.05) of microbial taxa in the ASL group than the ASH group, suggesting the community of microbes spanned a broader evolutionary range. The NPShannon and Shannon indices for the ASL group were also significantly higher (*p* < 0.05). In contrast, the Simpson index was significantly higher (*p* < 0.05) in ASH compared to ASL group.

**Fig. 6:**
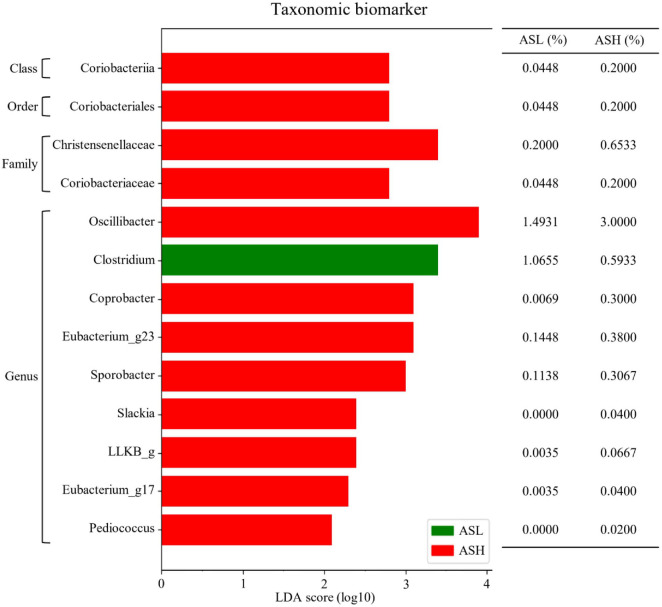
Taxonomic biomarkers for the ASL and ASH groups using linear discriminant analysis effect size (LEfSe). Microbiota patterns between ASL and ASH groups are analyzed using a linear discriminant analysis coupled with LEfSe. Results present differentially abundant microbiota taxa; red: ASL group, green: ASH group. ASH: Ankylosing spondylitis with high disease activity, defined as ASDAS-CRP ≥ 2.1; ASL: Ankylosing spondylitis with low disease activity, defined as ASDAS-CRP < 2.1.

Beta diversity analysis between the ASL and ASH groups using Jensen–Shannon, Generalized UniFrac, Bray–Curtis and UniFrac metrics revealed partial separation in the PCoA plots (Fig. 6B). However, no differences in statistical significance were observed in the PERMANOVA test in microbial composition among the groups across all distance metrics (*p* > 0.05; Fig. 6C), despite significant changes in microbial richness and diversity at the species level.

UPGMA hierarchical clustering based on Jensen–Shannon divergence, Bray–Curtis dissimilarity, Generalized UniFrac distance as well as UniFrac (Fig. S2.) indicated that distinct separation among the ASL and ASH groups was absent, indicating that the microbial community composition did not cluster clearly by disease severity.

### Taxonomic Biomarker Discovery (ASL vs. ASH)

LEfSe analysis with LDA identified differentially abundant taxa between the ASL and ASH groups (Fig. 6). Coriobacteriia was noticeably increased in the ASH group at the class level (LDA score ≥ 2.8), along with Coriobacteriales at the order level (LDA score ≥ 2.8). At the family level, Christensenellaceae (LDA score ≥ 3.4) and Coriobacteriaceae (LDA score ≥ 2.8) were significantly abundant in the ASH group. At genus level, *Oscillibacter* (LDA score ≥ 3.9), *Coprobacter* (LDA score ≥ 3.1), *Eubacterium_g23* (LDA score ≥ 3.1), and *Sporobacter* (LDA score ≥ 3.0) were significantly abundant in ASH group. Conversely *Clostridium* (LDA score ≥ 3.4) were significantly enriched in ASL.

### Analysis of the Correlation between Bacterial Genera and AS Disease Activity

**Fig. 7:**
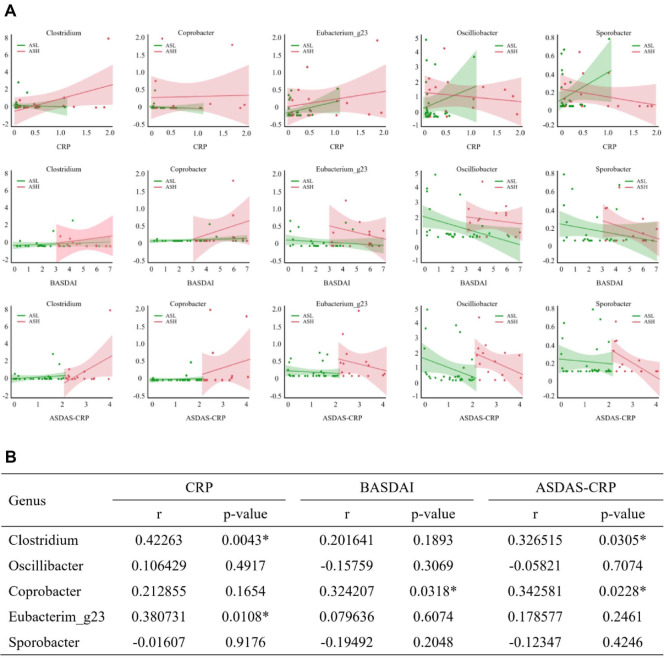
Correlations between bacterial genera abundance and disease activity. (**A**) Correlation analysis between bacterial genera and ankylosing spondylitis disease activity score: including C-reactive protein (CRP), Bath Ankylosing Spondylitis Disease Activity Index (BASDAI), and Ankylosing Spondylitis Disease Activity Score-CRP (ASDAS-CRP). (**B**) Pearson’s correlation coefficient analysis.

To determine the interdependence between the relative abundance of bacterial genera and disease activity parameters correlation analysis was conducted, including CRP, BASDAI, and ASDAS-CRP (Fig. 7A). In the ASH group, CRP positively correlated with *Clostridium*, *Coprobacter*, and *Eubacterium_g23* abundance, and negatively correlated with *Oscillibacter* and *Sporobacter* abundance. In the ASH group, BASDAI correlated with *Clostridium* and *Coprobacter* abundance positively while correlated with *Eubacterium_g23*, *Oscillibacter*, as well as *Sporobacter* abundance negatively. In the ASH group, ASDAS-CRP was positively associated with *Clostridium* and *Coprobacter* abundance while negatively proportionate to *Eubacterium_g23*, *Oscillibacter*, *Sporobacter* abundance.

Pearson correlation coefficient analysis assessed statistical significance (Fig. 7B). *Clostridium* abundance significantly correlated with CRP (*r* = 0.423, *p* = 0.004) and ASDAS-CRP (*r* = 0.327, *p* = 0.031) levels. *Coprobacter* abundance was closely tied to the BASDAI (*r* = 0.324, *p* = 0.032) and ASDAS-CRP (*r* = 0.343, *p* = 0.023). Additionally, *Eubacterium_g23* abundance significantly correlated with CRP (*r* = 0.381, *p* = 0.011).

## Discussion

This study investigated the gut microbiota characteristics of AS patients compared with healthy controls. AS patients exhibited a distinct gut microbial profile compared to healthy individuals. LDA and LEfSe identified Firmicutes, Clostridia, Clostridiales, Lachnospiraceae, Ruminococcaceae, and *Faecalibacterium* as being enriched in healthy controls. In contrast, Proteobacteria, Gammaproteobacteria, Enterobacterales, Enterobacteriaceae, and *Escherichia* spp. were enriched in patients with AS, representing potential biomarkers for identifying these patients. These differences in microbiome composition are consistent with previously reported findings [45, 46], suggesting that specific bacterial groups exert important effects on AS pathogenesis [47].

Gut microbiota produce metabolites known as short-chain fatty acids (SCFAs), such as butyrate, acetate, and propionate [48]. In this study, the relative abundances of SCFA-producing microbiota, including Ruminococcaceae, *Faecalibacterium*, Lachnospiraceae, and *Roseburia* [49] have been noticeably lower with AS patients comparing to HC. *Clostridia* spp. were also reduced in the AS group. This class of bacteria is critical in fermenting complex carbohydrates, such as dietary fiber, to generate SCFAs [50]. Butyrate produced by *Faecalibacterium prausnitzii* reduces CD4^+^IL-17A^+^ T cell differentiation and exhibits anti-inflammatory effects in animal models [51, 52]. SCFAs also provide energy for intestinal epithelial cells and enhance tight junction protein expression such as claudins as well as occludins, strengthening gut barrier and preventing leaky gut syndrome [53]. Conversely, Enterobacteriaceae and *Escherichia*, were significantly prolific in AS patients, produce pro-inflammatory molecules, including lipopolysaccharide. The loss of SCFA-producing taxa reduces butyrate-mediated inhibition of IL-17A+ T cell differentiation, potentially amplifying the IL-23/IL-17 axis [54]. At the same time, the overgrowth of *Enterobacteriaceae* and *Escherichia* may stimulate IL-6 and TNF-α production through LPS-driven immune responses [55]. Thus, the observed microbiome changes are mechanistically consistent with the IL-23/IL-17/IL-6/TNF-α axis emphasized in AS pathogenesis. Certain *Escherichia* species, particularly pathogenic *Escherichia coli*, can damage the intestinal mucosa and contribute to inflammatory bowel disease [56]. Higher frequencies of Th1 cells reacting to conserved *E. coli* proteins have been observed in the synovial fluid mononuclear cells of patients with AS, but not in those with rheumatoid arthritis, suggesting that *E. coli* acts as a mucosal marker of AS pathogenesis [57].

While alpha diversity metrics (diversity and species richness) show no statistically significant differences between the AS and HC groups, certain indices (ACE, Jackknife, and phylogenetic diversity) suggested a trend toward reduced richness in patients with AS, consistent with a previous study [46]. Within the AS cohort, higher disease activity (ASDAS-CRP ≥ 2.1) was linked to significantly lower alpha diversity. This supports the connections between AS disease activity, functional impairment, and gut dysbiosis [58, 59]. However, while beta diversity analyses revealed significant differences in the microbial community structure between the AS and HC groups, hierarchical clustering did not detect clear segregation between groups. This suggests that while the overall community composition differed, considerable inter-individual variability existed.

A subgroup analysis comparing patients with low and high disease activity revealed subtle differences. Although the overall taxonomic composition was similar, the ASH group demonstrated enrichment of *Oscillibacter* and loss of *Clostridium_g24*, suggesting that microbial imbalance may be exacerbated in more active disease states. Similarly, a higher prevalence of *Oscillibacter* has been detected juvenile idiopathic arthritis patients associated with the HLA-B27 gene [60]. Conversely, the gut microbiota of patients with IBD exhibits dysbiosis, with low *Oscillibacter* relative abundance [61]. *Oscillibacter* produces anti-inflammatory metabolites that reduce Th17 cell polarization, a pro-inflammatory T cell subset linked to autoimmune diseases [62]. Moreover, in metabolic syndrome models, *Oscillibacter* exacerbates fatty tissue inflammation associated with high-fat diets by activating pro-inflammatory pathways [63]. These findings underscore *Oscillibacter*'s dual role as an immune modulator and a context-dependent promoter of inflammation, warranting further investigation.

*Clostridium* was enriched in the ASL group. However, *Clostridium* abundance positively correlated with ASDAS-CRP and CRP levels. *Clostridium* species exhibit diverse effects on inflammation [64]. In low disease activity states, beneficial species, such as butyrate-producing *Clostridium leptum*, may proliferate and help suppress inflammation [65]. Conversely, under high disease activity, increased intestinal permeability may facilitate the adhesion and proliferation of pathogenic *Clostridium* spp. that can act as pro-inflammatory mediators [66]. Although butyrate generally possesses anti-inflammatory properties, it can also increase IL-23 production by stimulating dendritic cells, potentially exacerbating inflammatory responses [67].

*Coprobacter* positively correlated with disease activity in AS, as assessed by the BASDAI and ASDAS-CRP scores. *Coprobacter* spp. are obligate, anaerobic, gram-negative, rod-shaped bacteria originally isolated from human feces, with *Coprobacter fastidiosus* being the first species reported [68]. *Coprobacter* may contribute to gut barrier integrity through SCFA synthesis, modulating the immune response, and maintaining intestinal homeostasis [69]. Previous studies have identified potential biomarkers of AS disease activity. The abundances of *Dialister*, *Ruminococcus gnavus*, and *Roseburia* positively correlate with ASDAS and BASDAI [19, 20, 70]. BASDAI positively correlates with the relative abundance of *Escherichia* and *Klebsiella*, and negatively correlates with Lachnospiraraceae [71]. Although increased abundance of *Coprobacter* has been linked to neuropsychiatric disorders [71], its potential association with rheumatic diseases, including AS, has not yet been established. Hence, while *Coprobacter* may serve as a potential biomarker for AS disease activity, additional research is needed to elucidate their roles under these conditions.

This study has several limitations. First, temporal relationships between gut microbial composition and AS pathogenesis remain unclear in this cross-sectional study. Second, functional insights into microbial metabolism were not assessed. Third, potential confounding factors, such as medication use, diet, and lifestyle differences were not fully controlled. Besides, the absence of qPCR or culture-based validation for the proposed microbial biomarkers and covariate-adjusted sensitivity analyses poses a limitation of the study. Future longitudinal studies with functional metagenomic analyses are warranted to shed light on the mechanistic links among the human gut microbiota and AS.

In conclusion, this study verifies that AS patients harbor a distinct gut microbiome marked by an increased abundance of inflammation-promoting Proteobacteria and decreased abundance of beneficial Firmicutes. Lower microbial diversity relates to higher disease activity, and specific genera, including *Coprobacter*, may represent biomarkers of AS disease activity. These findings underscore significance of gut microbiota with AS pathogenesis while suggesting that microbiome-targeted interventions represent novel therapeutic strategies. Multi-omics as well as longitudinal studies are required to reconfirm the overall relationships of AS.

## Supplemental Materials

Supplementary data for this paper are available on-line only at http://jmb.or.kr.



## Figures and Tables

**Table 1:** Clinical characteristics of the study population.