# Supplementary material for: Alterations in the Gut Microbiome in Ankylosing Spondylitis and Their Correlation with Disease Activity
Source: J Microbiol Biotechnol. 2025 Dec 18;35:e2508043. doi: 10.4014/jmb.2508.08043 (PMC12740847; doi:10.4014/jmb.2508.08043)
Supplement: Supplementary file 1 [file jmb-35-e2508043-supple.pdf]

J. Microbiol. Biotechnol. 2025. 35: e2508043

https://doi.org/10.4014/jmb.2508.08043

Supplementary Table and Figures

Table S1. Clinical characteristics of AS patients according to disease activity.

	ASH (*n* = 15)	ASL (*n* = 29)	*p*-value
Age, years, mean ± SD	44.9 ± 14.9	41.2 ± 14.1	0.424
Male, *n* (%)	14 (93.3)	25 (86.2)	0.647
BMI, kg/m^2	27.4 ± 4.2	23.9 ± 3.8	0.006
Smoking, *n* (%)			0.445
Never	4 (26.7)	9 (31.0)	
Ex-smoker	4 (26.7)	12 (41.4)	
Current smoker	7 (46.7)	8 (27.6)	
Alcohol, *n* (%)			0.746
Never	4 (26.7)	7 (24.1)	
≤ 1 day/week	6 (40.0)	16 (55.2)	
2–3 days/week	4 (26.7)	4 (13.8)	
≥ 4 days/week	1 (6.6)	2 (6.9)	
Comorbidities, *n* (%)			
Hypertension	2 (13.3)	3 (10.3)	1.000
Heart disease	1 (6.7)	1 (3.4)	1.000
Diabetes	3 (20.0)	3 (10.3)	0.394
Liver disease	0 (0.0)	2 (6.9)	0.540
Cancer	1 (6.7)	2 (6.9)	1.000
Periodontitis	0 (0.0)	0 (0.0)	-
ESR, mm/h	21.8 ± 19.5	10.8 ± 14.1	0.037
CRP, mg/dL	0.72 ± 0.61	0.15 ± 0.20	0.003
AS manifestations, *n* (%)			
HLA-B27 positive	14 (93.3)	28 (96.6)	1.000
Uveitis	2 (13.3)	5 (17.2)	1.000
Inflammatory bowel disease	1 (6.7)	1 (3.4)	1.000
Peripheral arthritis	5 (33.3)	8 (27.6)	0.692
Disease duration, months	67.3 ± 54.0	60.6 ± 51.8	0.687
Disease activity			
BASDAI, mean ± SD	4.77 ± 1.28	2.35 ± 1.73	< 0.001
VAS, mean ± SD	5.33 ± 1.80	1.96 ± 1.84	< 0.001
BASFI, mean ± SD	2.83 ± 2.15	0.82 ± 1.17	< 0.001
ASDAS-CRP, mean ± SD	2.85 ± 0.59	1.21 ± 0.65	< 0.001
ASDAS-ESR, mean ± SD	2.97 ± 0.79	1.56 ± 0.76	< 0.001
Medications, *n* (%)			
NSAID	12 (80.0	19 (65.5)	0.488
Sulfasalazine	6 (40.0)	10 (34.5)	0.718
Methotrexate	0 (0.0)	2 (6.9)	0.540

Glucocorticoid	1 (6.7)	3 (10.3)	1.000
Anti-TNF	6 (40.0)	11 (37.9)	0.894

AS, ankylosing spondylitis; ASDAS, ankylosing spondylitis disease activity score; ASH: Ankylosing spondylitis with high disease activity, defined as ASDAS-CRP ≥ 2.1; ASL: Ankylosing spondylitis with low disease activity, defined as ASDAS-CRP < 2.1; BASDAI, Bath Ankylosing Spondylitis Disease Activity Index; BASFI, Bath Ankylosing Spondylitis Functional Index; BMI, body mass index; CRP, C-reactive protein; ESR, erythrocyte sedimentation rate; HLA, human leukocyte antigen; NSAID, non-steroidal anti-inflammatory drug; SD, standard deviation; TNF, tumor necrosis factor; VAS, visual analog scale

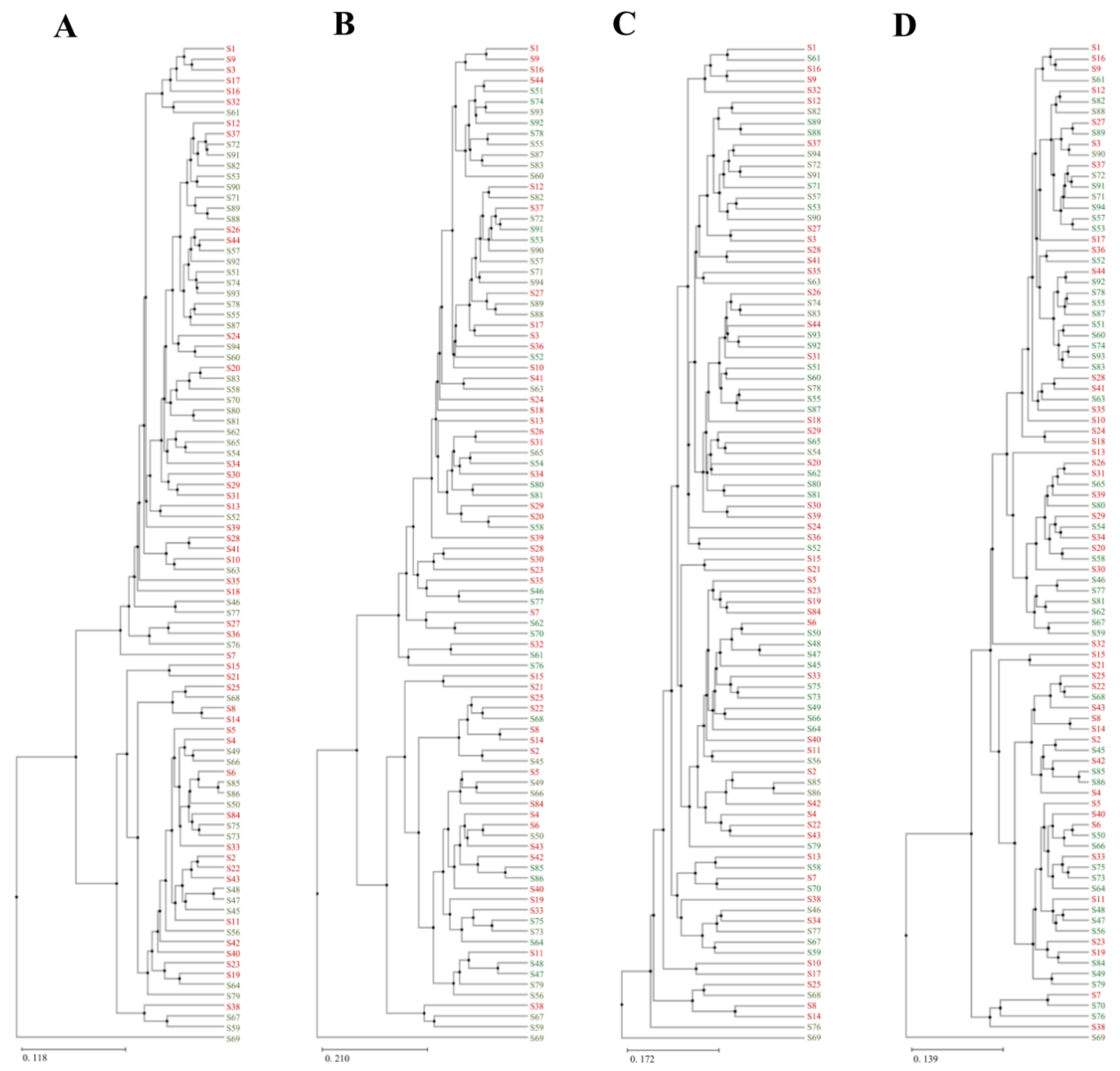

Fig. S1. Clustering using the Unweighted Pair Group Method with Arithmetic Mean (UPGMA). Healthy controls (HC) and patients with ankylosing spondylitis (AS) are analyzed by (**A**) Jensen-Shannon, (**B**) Bray–Curtis, (**C**) Generalized UniFrac, and (**D**) UniFrac.

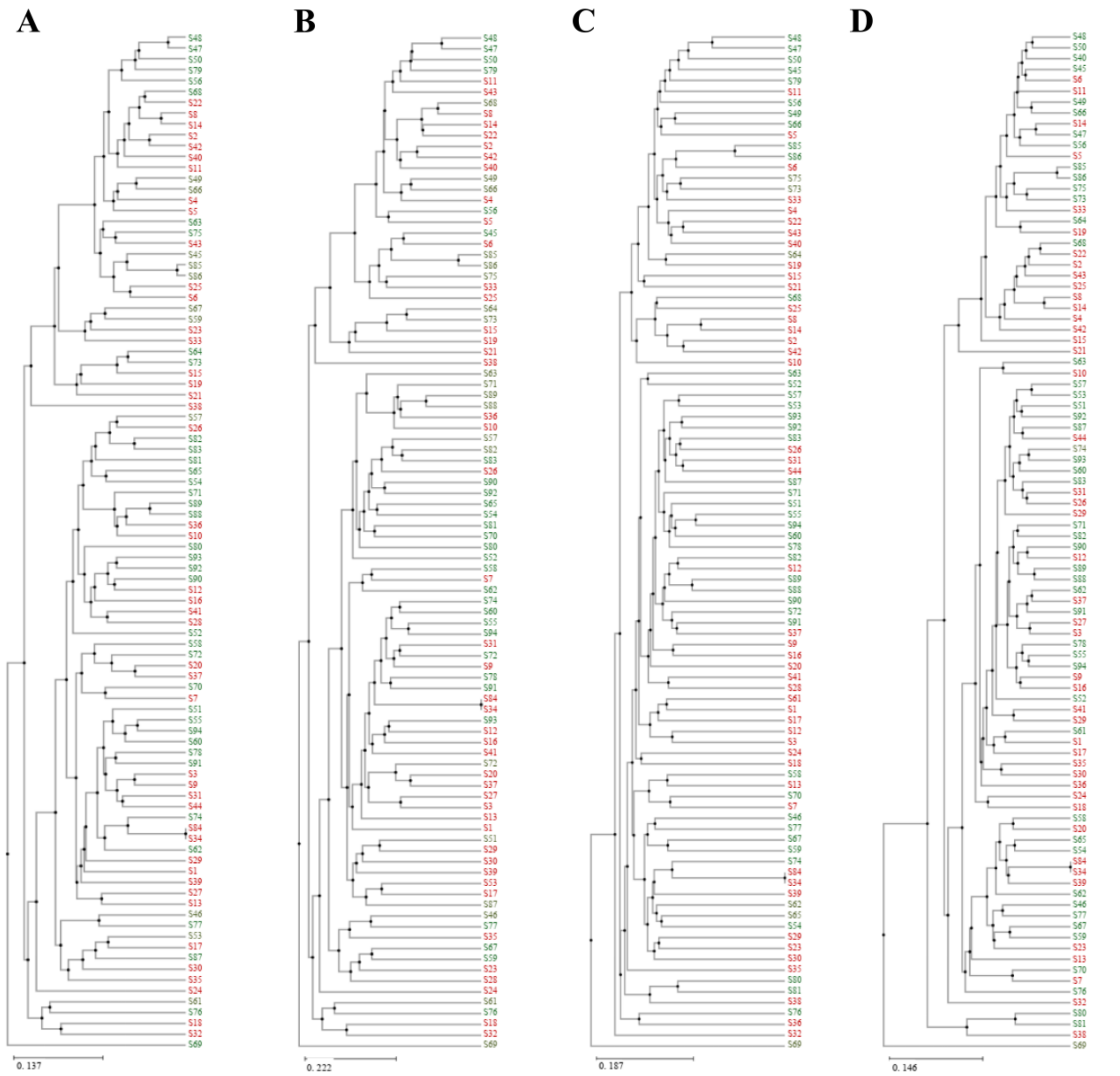

Fig. S2. Clustering using the Unweighted Pair Group Method with Arithmetic Mean (UPGMA). ASL and ASH groups analyzed by (**A**) Jensen-Shannon, (**B)** Bray–Curtis, (**C**) Generalized UniFrac, and (**D**) UniFrac. ASH: Ankylosing spondylitis with high disease activity, defined as ASDAS-CRP ≥ 2.1; ASL: Ankylosing spondylitis with low disease activity, defined as ASDAS-CRP < 2.1

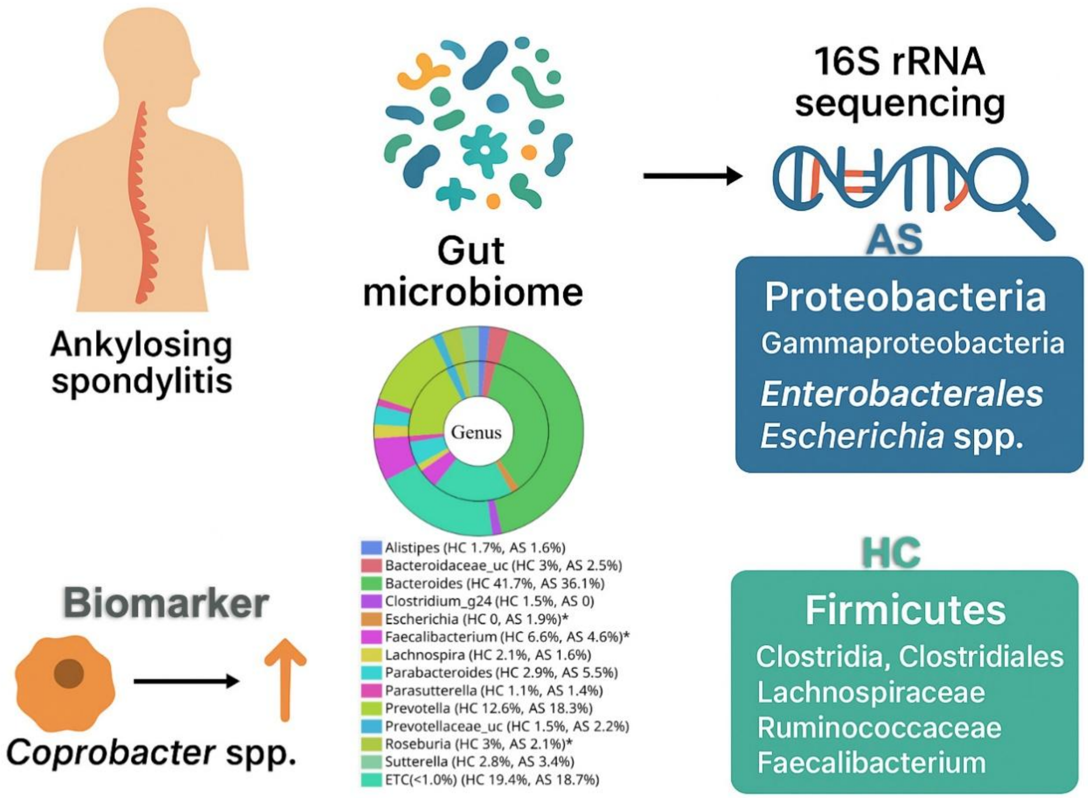

Fig. S3. Graphical abstract of Gut Microbiome Alterations and Biomarker Identification in Ankylosing Spondylitis.
